# A Scientific Rationale for Using Cystic Fibrosis Transmembrane Conductance Regulator Therapeutics in COVID-19 Patients

**DOI:** 10.3389/fphys.2020.583862

**Published:** 2020-11-04

**Authors:** Darcy Lidington, Steffen-Sebastian Bolz

**Affiliations:** ^1^Department of Physiology, University of Toronto, Toronto, ON, Canada; ^2^Toronto Centre for Microvascular Medicine at The Ted Rogers Centre for Heart Research Translational Biology and Engineering Program, University of Toronto, Toronto, ON, Canada; ^3^Heart and Stroke/Richard Lewar Centre of Excellence for Cardiovascular Research, University of Toronto, Toronto, ON, Canada

**Keywords:** tumor necrosis factor, lung, immune cells, brain, cerebrovascular

## Abstract

Several pathological manifestations in coronavirus disease 2019 (COVID-19), including thick mucus, poor mucociliary clearance, and bronchial wall thickening, overlap with cystic fibrosis disease patterns and may be indicative of “acquired” cystic fibrosis transmembrane conductance regulator (CFTR) dysfunction. Indeed, tumor necrosis factor (TNF), a key cytokine driving COVID-19 pathogenesis, downregulates lung CFTR protein expression, providing a strong rationale that acquired CFTR dysfunction arises in the context of COVID-19 infection. In this perspective, we propose that CFTR therapeutics, which are safe and generally well-tolerated, may provide benefit to COVID-19 patients. Although CFTR therapeutics are currently only approved for treating cystic fibrosis, there are efforts to repurpose them for conditions with “acquired” CFTR dysfunction, for example, chronic obstructive pulmonary disease. In addition to targeting the primary lung pathology, CFTR therapeutics may possess value-added effects: their anti-inflammatory properties may dampen exaggerated immune cell responses and promote cerebrovascular dilation; the latter aspect may offer some protection against COVID-19 related stroke.

## Introduction

The 2019 *severe acute respiratory syndrome coronavirus 2* (SARS-CoV-2) outbreak has taken a substantive toll on world health. The resulting illness, *coronavirus disease 2019* (COVID-19), primarily manifests as a respiratory pathology; in severe cases, a “cytokine storm” and severe acute respiratory syndrome ensues (Huang et al., 2020). Since the pathophysiology of related coronavirus infections (e.g., *severe acute respiratory syndrome coronavirus*, SARS-CoV and *Middle East respiratory syndrome coronavirus*, MERS-CoV) is not completely understood (Huang et al., 2020), our insights and capabilities to effectively treat both the primary lung injury and remote organ pathologies that arise as a result of SARS-CoV-2 infection are limited. It is therefore incumbent that we accelerate clinical trials for any readily available and acceptably safe medications that may reduce mortality and/or morbidity.

The majority of repurposed medications under investigation to date are designed to disrupt viral entry or replication (e.g., lopinavir, ribavirin, and remdesivir); the remaining medications under study are primarily anti-inflammatory in nature (e.g., corticosteroids and anti-cytokine therapeutics Sanders et al., 2020). Anti-inflammatory medications are not currently recommended as an intervention, as previous experience indicates that they delay viral clearance and are not associated with improved mortality (Huang et al., 2020). In lieu of interfering with the production of inflammatory mediators and/or interfering with specific inflammatory entities, there may be significant opportunities to improve outcomes in COVID-19 patients by modulating important downstream effectors of these inflammatory signals.

Tumor necrosis factor (TNF) is a central inflammatory mediator that is intimately linked to COVID-19 by virtue of the SARS-CoV-2 infection mechanism (i.e., the activation of TNF alpha converting enzyme; TACE; Haga et al., 2008) and through its systemic presence in more severe cases (Huang et al., 2020). Thus, TNF signaling is an early and persistent aspect of the COVID-19 disease progression. Of the many effects TNF signaling has, one intriguing effect is a broad downregulation of cystic fibrosis transmembrane conductance regulator (CFTR) expression in multiple tissues, most notably, in the lung and brain (Meissner et al., 2012; Yagi et al., 2015). CFTR is widely expressed in many critical organs, including the lung, brain, heart, kidney, gut, pancreas, immune cells, and blood vessels (Meissner et al., 2012; Yagi et al., 2015; Castellani and Assael, 2017; Lidington et al., 2019; Lara-Reyna et al., 2020): it is therefore tempting to propose that reduced CFTR activity mediates several deleterious TNF effects. This would place CFTR-modifying therapeutics in a prime position to alter the course and severity of the COVID-19 pathology. In this regard, we propose that CFTR therapeutics could improve lung function, positively influence immune cell activity, and reduce ischemia-dependent neurological symptoms.

## CFTR and the Lung

The cystic fibrosis transmembrane conductance regulator is best known for its central role in lung mucociliary clearance, which is best illustrated by the profound pulmonary manifestations that arise in cystic fibrosis (Boucher, 2007). The core function of CFTR in this context is to maintain proper hydration of both the secreted mucous and the less viscous peri-ciliary layer that resides underneath it (Livraghi and Randell, 2007; Collawn and Matalon, 2014). Even modest osmotic perturbation yields highly viscous mucous and a compression of the peri-ciliary layer that slows mucus transport (Anderson et al., 2015; Hill et al., 2018). The inability to effectively clear mucus results in airway obstruction (i.e., physical plugging of the airways) and inflammation, even in the absence of bacterial infection (Livraghi-Butrico et al., 2012). In addition to mucociliary clearance, CFTR also modulates bronchiolar constriction. Bronchial hyperreactivity is a common feature of cystic fibrosis and targeting bronchiolar smooth muscle cells with CFTR activators is capable of inducing relaxation symptoms (McCuaig and Martin, 2013; Norez et al., 2014). Again, much of the research in this field focuses on mutated CFTR and cystic fibrosis; however, even a relatively mild inflammatory setting clearly downregulates wild-type CFTR expression in the lung (Meissner et al., 2012).

The lung is the primary target of SARS-CoV-2 infection and has received the lion’s share of attention. We know that bronchial wall thickening, which is indicative of obstruction and inflammatory damage, is highly prevalent in COVID-19 patients, especially those with severe pulmonary symptoms (65%; Li et al., 2020). Air bronchograms commonly display opaque, airless lung regions that are presumed to be filled with inefficiently cleared mucus (Ye et al., 2020). This latter presumption requires more autopsy data than are currently available to confirm; however, at present, there is at least one autopsy report confirming the presence of viscous mucous and fibrosis in a COVID-19 patient (Liu et al., 2020). All of these manifestations are hallmarks of cystic fibrosis and could arise from or be aggravated by deficient CFTR activity: in this context, it is intriguing to hypothesize that a substantial subset of COVD-19 cases would benefit from the use of cystic fibrosis medications.

## CFTR and Immune Responses

The importance of CFTR in the regulation of immune cell physiology is quickly emerging. As reviewed by Lara-Reyna et al. (2020), several immune cell types, including neutrophils, monocytes, and macrophages, are profoundly affected by the loss of CFTR function. As specific examples, the absence of CFTR appears to (i) shift neutrophils and macrophages into a more activated state (Adib-Conquy et al., 2008; Zhang et al., 2018), (ii) augment both basal and stimulated cytokine release (Bonfield et al., 2012; Zhang et al., 2018; Lara-Reyna et al., 2020), (iii) hamper infection resolution (Bonfield et al., 2012), and (iv) impair the production of agents that destroy phagocytosed pathogens (e.g., hypochlorous acid and reactive oxygen species; Zhou et al., 2013; Zhang et al., 2018). A common thread across these traits is that CFTR serves a strong anti-inflammatory role in multiple immune cell types. This prompts two pertinent questions: (i) do inflammatory settings downregulate immune cell CFTR and (ii) would enhancing CFTR activity during inflammatory responses confer benefit? Since most studies investigating to role of CFTR in inflammation focus on mutated CFTR and cystic fibrosis, the data available from settings with wild-type CFTR are sparse. Nevertheless, a study by Li et al. (2017), who investigated macrophage activity in inflammatory atherosclerotic plaques, suggests that the answer might be “yes” in both cases. Data from cystic fibrosis patients help reinforce this conclusion, as taking CFTR therapeutics has positive effects on the functional characteristics of their immune cells (Zhang et al., 2018). It is tempting, therefore, to speculate that, in addition to its direct effects on lung function, CFTR therapeutics may have a “value-added effect” of dampening exaggerated immune responses that damage the lungs of COVID-19 patients.

## CFTR and the Brain

The cystic fibrosis transmembrane conductance regulator is widely expressed in the brain (Guo et al., 2009) and is a prominent regulator of cerebral artery vasoconstriction (Lidington et al., 2019): it is therefore poised to play a modulatory role in ischemic brain injury. In cerebral arteries, CFTR mediates a strong vasodilatory influence by sequestering pro-constrictive molecules away from their receptors through its transporter function (Meissner et al., 2012). In inflammatory settings, microvascular CFTR expression is downregulated, leading to a pro-constrictive phenotype (Meissner et al., 2012; Lidington et al., 2019). Preclinical data from a mouse model of subarachnoid hemorrhage, a highly injurious cerebral pathology with a strong inflammatory component, clearly demonstrate that restoring CFTR expression/activity improves cerebral perfusion, reduces neuronal injury, and protects cognitive function (Lidington et al., 2019). Since CFTR expression appears to be largely restricted to the cerebrovascular microcirculation, one key advantage of this therapeutic strategy over traditional vasodilators is that cerebral perfusion can be increased without a significant impact on mean arterial pressure or other systemic hemodynamic parameters (Lidington et al., 2019).

Non-specific neurological manifestations, such a confusion, headache, and dizziness are commonly reported symptoms for COVID-19; however, peer-reviewed literature examining the incidence and extent of neurological symptoms is presently scarce. According to a recent meta-analysis on the topic (Asadi-Pooya and Simani, 2020), most of the available data are low quality and remarkably, only one study has specifically addressed this issue. This particular study by Mao et al. (2020) shows that neurological symptoms are common in COVID-19 (36%); in patients with more severe cases, based on respiratory status, the incidence is even higher (46%). Patients who survive acute respiratory distress syndrome are frequently left with neurological deficits (Sasannejad et al., 2019): these individuals undoubtedly suffered from ischemic injury caused by reduced cerebral perfusion, which is a well-known complication of systemic inflammatory responses, for example sepsis (Burkhart et al., 2010). In addition to improving neurological symptoms, increasing cerebral perfusion may offer some protection against COVID-19 related stroke. Strokes are emerging as a serious complication in COVID-19 that is generally attributed to the remarkably high incidence of thrombotic complications (Hess et al., 2020; Klok et al., 2020). Increasing cerebral perfusion would be expected to reduce one predilecting factor for coagulation (i.e., low blood flow) and reduce stroke severity in the event of a cerebral thrombosis. Thus, it is intriguing to propose that CFTR therapeutics could represent a safe means of improving neurological symptoms, reducing the incidence and severity of strokes, and minimizing long-term cognitive impact in COVID-19 patients.

## CFTR Therapeutic Repurposing for Acquired CFTR Dysfunction

Cystic fibrosis transmembrane conductance regulator medications are currently only approved for treating cystic fibrosis; however, there is significant interest in repurposing them for patients with “acquired” CFTR dysfunction (Peckham et al., 2020), most notably smokers with chronic bronchitis and patients with severe viral lung infections. With regard to the first group, cigarette smoke has been shown to reversibly depress both pulmonary and non-pulmonary CFTR activity in mice (Raju et al., 2013). Although pulmonary CFTR activity has not been assessed in human smokers, cigarette smoke clearly reduces non-pulmonary CFTR activity in humans, as evidenced by sweat chloride levels and nasal potential differences (Sloane et al., 2012; Raju et al., 2013). Subsequent *in vitro* studies demonstrated that the CFTR therapeutic ivacaftor significantly improves airway cell function following perturbation with smoke extract (Sloane et al., 2012; Raju et al., 2016). With this solid foundation, Solomon et al. (2016) completed a pilot evaluation of ivacaftor in smokers with chronic obstructive pulmonary dysfunction (COPD): although the trial was underpowered and found no statistically significant effect, the authors reported that improvements in CFTR activity and respiratory symptoms were evident and encouraged a phase 2 trial that is currently underway (NCT03085485).

The latter example is based on the finding that severe influenza infections negatively impact pulmonary surfactant secretion, setting the stage for secondary infection, inflammation, and compromised respiratory function (reviewed by Londino et al., 2017). As a component of the influenza virus pathology, CFTR may be targeted for rapid degradation (Londino et al., 2013, 2015; Brand et al., 2018). The CFTR therapeutic lumacaftor can stabilize CFTR against degradation (Okiyoneda et al., 2013; Lidington et al., 2019): its use in experimental settings restores airway cell function following *in vitro* influenza infection (Brand et al., 2018). Although these data provide another strong foundation for clinical testing, to our knowledge, this potential application has yet to be tested in the clinical arena.

## Caveats to CFTR Therapeutic Use

Although there is a reasonable basis to propose that CFTR dysfunction occurs in COVID-19, several caveats apply. First and foremost, deficient CFTR activity has not been established in COVID-19 and the net effect of a CFTR deficiency in a complex response involving multiple cytokines and cell types remains to be characterized. Similarly, there is a large translational gap between animal models and human subjects and thus, benefits supported by animal data are not necessarily realized in a clinical setting. This is pertinent to several new therapeutic indications currently under investigation, including the cerebrovascular effects of CFTR therapeutics described above: there may be clear benefits evident in mice, but these await confirmation in a human setting. Finally, the available CFTR therapeutics (e.g., Kalydeco, Orkambi, and Trikafta) are prohibitively expensive, an unfortunate fact that may deter their widespread use.

## Outlook

Cystic fibrosis transmembrane conductance regulator therapeutics are generally considered safe and are well tolerated. Given the dire mortality and morbidity statistics for COVID-19, we believe that there is sufficient evidence to support initiating proof of concept clinical trials addressing the potential benefit of CFTR therapeutics in patients with COVID-19. We propose initiating treatment immediately upon hospital admission, since CFTR dysfunction is likely an early and persistent aspect of the COVID-19 disease progression and therefore likely to be present at the time of diagnosis. If safety and efficacy criteria are adequately met, treating COVID-19 patients who do not require hospitalization, especially those who are at high risk (e.g., older individuals or those with pre-existing conditions, such as diabetes), could be considered.

## Author Contributions

DL and S-SB contributed equally to the literature research, writing, and editing of this article. Both the authors contributed to the article and approved the submitted version.

### Conflict of Interest

DL and S-SB are consultants for Qanatpharma GmbH; neither have received remuneration or consulting fees from Qanatpharma. S-SB is an executive board member of Aphaia Pharma AG and DL is a paid consultant for Aphaia Pharma AG. Neither Qanatpharma GmbH nor Aphaia Pharma AG had any financial or intellectual involvement in this article.
